# The antipsychotic drug lurasidone inhibits coronaviruses by affecting multiple targets

**DOI:** 10.3389/fcimb.2024.1487604

**Published:** 2024-11-25

**Authors:** Sara Baroni, Tea Carletti, Manuela Donalisio, Irene Arduino, Irene Cazzaniga, Toni Giorgino, Francesca Esposito, Alessia Porta, Luisa Diomede, Ada De Luigi, Marco Gobbi, David Lembo, Alessandro Marcello, Enzo Tramontano, Mario Milani, Eloise Mastrangelo

**Affiliations:** ^1^ Department of Molecular Biochemistry and Pharmacology, Istituto di Ricerche Farmacologiche Mario Negri Istituto di Ricovero e Cura a Carattere Scientifico (IRCCS), Milano, Italy; ^2^ Laboratory of Molecular Virology, International Centre for Genetic Engineering and Biotechnology, Trieste, Italy; ^3^ Dipartimento di Scienze Cliniche e Biologiche, Università di Torino, Regione Gonzole, Turin, Italy; ^4^ Dipartimento di Bioscienze, Università degli Studi di Milano, Milano, Italy; ^5^ Consiglio Nazionale delle Ricerche, Istituto di Biofisica, Milano, Italy; ^6^ Department of Life and Environmental Sciences, University of Cagliari, Monserrato, Italy

**Keywords:** coronaviruses, papain-like protease, Spike protein, ACE2 interaction, dual-target compound

## Abstract

Coronaviruses (CoVs) share key genomic elements critical for viral replication, suggesting the feasibility of developing therapeutics with efficacy across different viruses. In a previous work, we demonstrated the antiviral activity of the antipsychotic drug lurasidone against both SARS-CoV-2 and HCoV-OC43. In this study, our investigations on the mechanism of action of lurasidone suggested that the drug exhibits antiviral activity by targeting the papain-like protease (PL-Pro) of both viruses, and the Spike protein of SARS-CoV-2, thereby hampering both the entry and the viral replication. *In vitro* assays demonstrate that lurasidone significantly reduces viral load in infected cells, showing that the drug is a promising candidate for further development as a dual-action antiviral, offering a potential new strategy in the fight against COVID-19 and other coronavirus-related diseases.

## 1 Introduction

Novel emerging RNA viruses present a continuous threat to humankind; their evolution can be largely attributed to genetic variants of zoonotic viruses from animal reservoirs (Dharmarajan et al., 2022). Due to high mutation frequency, it is to be expected that new variants of RNA viruses will continuously emerge from the large natural pool. Increasing human population density combined with higher mobility, commercial transport, land exploitation and climate change, all contribute to exacerbating this picture. Today, we are unable to predict which virus may spread in the future: preparedness to meet such a threat depends on continuous research to enrich our knowledge on the molecular basis of viral infectivity and to promote the ability to take the necessary measures for viral control, including the prompt development of vaccines as well as the discovery of effective antiviral drugs.

Among RNA viruses, coronaviruses (CoVs) represent a large family of viruses that cause respiratory and gastrointestinal illnesses of varying severity. The CoV family is divided into four genera (from *alpha* to *delta*), and thus far human CoVs are limited to the *alpha* (HCoV-229E and HCoV-NL63) and *beta* genera (HCoV-OC43, HCoV-HKU1, SARS-CoV-1, SARS-CoV-2 and MERS-CoV). In terms of outcome, while most cases of SARS-CoV-2 infections display none to mild symptoms, some progress to pneumonia and multi-organ failure with a fatality rate estimated between 1% and 5%. By contrast, HCoV-OC43, HCoV-NL63 and HCoV-229E strains are considered low-pathogenic CoVs, endemic in humans, settling mainly in the upper respiratory tract and causing minor symptoms, although acute infections in infants, elderlies and immunocompromised patients may progress to severe disease requiring hospitalization.

Although Paxlovid (Niraj et al., 2022) and Molnupiravir (Teli et al., 2023) have been identified as promising anti-SARS-CoV-2 agents, there is still a need for new antivirals possibly active against different emerging variants [i.e. the very recent FLiRT variants (Kumar et al., 2024)]. Despite their species diversity, CoVs share key genomic elements that are essential for viral replication, suggesting the possibility of designing broad-spectrum therapeutics. Broad-spectrum drugs are gaining attention as antivirals for their potential to quickly curb the spread of infections, offering a rapid response while specific treatments or vaccines are still in development (Geraghty et al., 2021).

After the release of the viral RNA genome into the cytoplasm, the main protease (Mpro) and the papain-like protease (PL-Pro) cleave the newly translated polyproteins, pp1a and pp1ab, into individual non-structural proteins (Nsps). The key actors of the viral replication-transcription complex (RTC) are the Helicase (Nsp13) and the RNA-dependent RNA Polymerase (RdRp, Nsp12), involved, respectively, in unwinding the viral RNA during replication and in synthesizing the new viral RNA genome (Mishchenko and Ivanisenko, 2022).

In a previous work, starting from an *in silico* docking on SARS-CoV-2 Nsps, we selected the antipsychotic drug lurasidone demonstrating its antiviral activity against both SARS-CoV-2 and HCoV-OC43 (Milani et al., 2021).

In this work we investigated the mechanism of action of the selected drug. Time of drug addition (ToA) experiments on SARS-CoV-2 indicated that lurasidone can inhibit viral replication when co-administered with the virus and during the early phase post-infection, when the Nsps are functionally active.

By using pseudotyped lentiviral vectors exposing the SARS-CoV-2 Spike protein, we demonstrated that lurasidone inhibits the viral entry and its efficacy is related to the specific Spike isoform. In contrast, lurasidone did not affect the entry of HCoV-OC43 that employs sialoglycan-based receptors on cell surface (Owczarek et al., 2018). We show that the strong inhibition of both SARS-CoV-2 and HCoV-OC43 during the early phase post-infection is likely dependent on a conserved Nsp, that we have identified as the PL-Pro.

Since viruses can develop resistance to treatments targeting a single component, a multi-targeted approach can hinder their capability to adapt and survive. In this contest, lurasidone, by targeting two critical components of the viral life cycle, such as PL-Pro and Spike-ACE interface, affects SARS-CoV-2 replication reducing the likelihood of drug resistance.

## 2 Materials and methods

### 2.1 Pseudotyped lentiviral vectors assays

#### 2.1.1 Cells

Human embryonic kidney (HEK) 293 cells expressing human receptor ACE2 (HEK293-ACE2) (Diomede et al., 2021) were maintained in Dulbecco modified Eagle Medium (DMEM; Gibco/Euroclone #ECB7501L) containing 10% heat-inactivated fetal bovine serum (FBS, Gibco #10270), L-glutamine (Gibco, #25030-024), non-essential amino acids (Gibco/Euroclone, #ECB3054D), and penicillin/streptomycin (Corning, #20-002-Cl). HEK293-ACE2 required puromycin (Genespin, Milano, Italy). Cells were cultured in T25 flasks at 37°C in a humidified 5% CO_2_ and routinely split every 4–5 days.

#### 2.1.2 Cell viability

HEK293-ACE2 cells were seeded (2 x 10^4^ cells/well) on 96-well plates in a complete DMEM medium with 10% FBS. After incubation for 24 hours at 37°C in humidified 5% CO_2_, the medium was replaced with a fresh one containing lurasidone previously dissolved in Milli-Q water at 0.1 – 100 µM. Control cells were treated with an equivalent volume of vehicle only. Cells were incubated for 24 hours at 37°C in humidified 5% CO_2_, then the medium was replaced with a fresh one without lurasidone. After an additional incubation of 24 hours at 37°C in humidified 5% CO_2_, HEK293-ACE2 cells were treated with 5 mg/ml 3-(4,5-dimethylthiazol-2-yl)-2,5 diphenyltetrazolium bromide (MTT) (Sigma Aldrich, St. Louis, MO, USA, #M5655-1G) in 5 mM Phosphate Buffered Saline (PBS), pH 7.4. After incubation for 4 hours at 37°C, MTT was removed, and the cells resuspended in isopropanol containing 0.04 M HCl. The absorbance of the samples was determined at 560 nm using a spectrophotometer (Infinite M200, Tecan, Männedorf, Switzerland), and the cell viability was expressed as a percentage of Vehicle-treated cells.

#### 2.1.3 Transduction assay

HEK293-ACE2 cells were seeded (2 x 10^4^ cells/well) on 96-well plates in a complete DMEM medium with 10% FBS. After 24 hours at 37°C in humidified 5% CO_2_, the medium was replaced with a fresh medium containing 1 – 5 µM lurasidone dissolved in Milli-Q water. Control cells were treated with the same volume of vehicle only. Cells were then incubated for 4 hours at 37°C in humidified 5% CO_2_ and then infected, in the presence of 10 µg/ml Polybrene (VectorBuilder, USA) with 6.25 - 50 MOI lentiviral vector exposing the SARS-CoV-2 Spike protein as surface glycoprotein, in the Wuhan, B.1.1.7 UK or B.1.351 SA isoform (VectorBuilder, USA) and eGFP as gene reporter. Cells infected with lentivirus not expressing Spike protein on the Envelope (Bald), not infected and non-drug treated (Vehicle) cells were employed as controls. The day after the transduction, the medium was replaced with a fresh one, and after additional 24 hours incubation at 37°C in humidified 5% CO_2_ the transduction efficiency was checked by determining the percentage of cells expressing GFP, using a ZOE™ fluorescent cell imager (Bio-Rad, Hercules, CA, USA). The ZOE™ images were analyzed with Fiji software, an open-source platform for biological-image analysis (Diomede et al., 2021). The transduction efficiency was expressed as the percentage of cells expressing GFP-fluorescent signal.

#### 2.1.4 Western blot analysis

HEK293-ACE2 cells were seeded (2.4 x 10^5^ cells/well) on 12-well plates in complete DMEM medium with 10% FBS and incubated for 24 hours at 37°C in humidified 5% CO_2_. The medium was then replaced with a fresh one containing 5 µM lurasidone. Control cells were treated with an equivalent volume of vehicle only. After incubation for 3, 6, and 24 hours at 37°C in humidified 5% CO_2_ the medium was removed, the cells collected and lysed for 15 min at 4°C with 20 mM Tris-HCl, pH 7.5, containing 150 mM NaCl, 1 mM Na_2_EDTA, 1 mM EGTA, 1% NP-40, 1% sodium deoxycholate, 2.5 mM sodium pyrophosphate, 1 mM β-glycerophosphate, 1 mM Na_3_VO_4_ and 1 µg/ml leupeptin. Samples were centrifuged for 10 min at 16,100 x *g* and the protein content was quantified by using a BCA protein assay kit (Thermofisher, Rockford, USA). 10 µg of total proteins were loaded in each lane, immunoblotted using 10% bis-Tris gel (Invitrogen, Waltham, MA, USA), and transferred to a PVDF membrane (Millipore, Vimodrone, Milan, Italy). The membranes were incubated overnight at 4°C with an anti-ACE2 AC18Z mouse monoclonal antibody (1:200, Santa Cruz Biotechnology, Santa Cruz, CA, USA) or anti-β-actin mouse monoclonal antibody (1:5000, Sigma Aldrich). Peroxidase-conjugated anti-mouse IgG (1:5000, Sigma Aldrich) was used as the secondary antibody. Hybridization signals were detected with a ChemiDoc XRS Touch Imaging System (Bio-Rad).

#### 2.1.5 Surface plasmon resonance direct binding assay

Recombinant human ACE2-Fc tag (rhACE2) (Acrobiosystem) at 5 μg/mL in PBST was captured on a HC30M (Xantec) sensor chip after the immobilization of anti-Fc antibody (Biorad) at 25 μg/mL in acetate buffer pH 5.0 by classic amine coupling chemistry on a NHS/EDC activated surface, reaching a final immobilization level of ~ 1000 RU. Simultaneously, a mix of IgG antibodies at 25 μg/mL in acetate buffer pH 5.0 was immobilized on a parallel surface and used as reference.

To test the direct binding of rhACE2 and lurasidone, the drug was injected at 10 µM in PBST 0.1% DMSO for 200 seconds at 30 μl/min on both surfaces. In a different channel, we also flowed SARS-CoV-2 Spike protein RBD (Wuhan isotype) at 10 nM in PBST as a positive control.

### 2.2 SARS-CoV-2 cell-based assays

#### 2.2.1 Cell lines and viruses

Vero E6 cells (ATCC-1586) and the human hepatocarcinoma Huh7 previously engineered to overexpress the human ACE2 receptor (Huh7-ACE2) (Milani et al., 2021) were cultured in Dulbecco’s Modified Eagle Medium (DMEM, Gibco) supplemented with 5% heat inactivated fetal bovine serum (FBS, Gibco). Huh7-hACE2 required puromycin (1 μg/ml, Gibco). Working stocks of SARS-CoV-2 ICGEB-FVG_5 (Licastro et al., 2020) isolated in Trieste, Italy, were routinely propagated and titrated on Vero E6 cells.

#### 2.2.2 Plaque assay

The viral titers were measured by Plaque assay. Briefly, Vero E6 (6 x 10^4^ cells/well) were seeded in 48-well plates in DMEM/10%FBS medium and the day after incubated with the harvested supernatants (serially diluted) for 1 h. The medium was then removed and, after a washing step with PBS, DMEM/2% FBS/1.5% CMC was added to each well. Cells were then fixed with 3.7% PFA solution at 72 h.p.i and then colored with a 1% Crystal Violet solution. Plaques were then counted, and the viral titers expressed as plaques forming units per ml (PFU/mL).

#### 2.2.3 Virus inactivation assay

The direct virucidal activity of lurasidone against SARS-CoV-2 was tested by incubating 300 PFU of SARS-CoV-2 with the drug at a concentration of 5 μM for 2 h at room temperature. The virus-drug mixture was subsequently diluted 1:10 and titrated by Plaque Assay.

#### 2.2.4 Time-of-addition assay

The antiviral activity of lurasidone when administered before, during or after SARS-CoV-2 infection was evaluated with time-of-addiction experiments. Huh7-ACE2 cells were seeded (6 x 10^4^ cells/well) on 12-well plates in DMEM medium. 24 hours later Huh-7-ACE2 cells were treated for 1 hour with 5 µM of lurasidone, washed and then infected at MOI 1 with SARS-CoV-2 (pre-treatment). Alternatively, the drug was added together with the virus at the time of infection and removed when the virus inoculum was washed from the cells (co-treatment). Finally, the drug was added on infected cells at 1, 4, 18 hours post infection (h.p.i.) and left in the solution until the end of the experiment (post-infection treatment). At 24 h.p.i. all supernatants were collected, and virus titrated by Plaque Assay.

### 2.3 HCoV-OC43 cell-based assays

#### 2.3.1 Cell lines and viruses

Human lung fibroblast cells MRC-5 (ATCC^®^ CCL-171) were grown in Dulbecco’s Modified Eagle Medium (DMEM; Sigma-Aldrich, St. Louis, MO, USA) supplemented with 1% (v/v) penicillin/streptomycin solution (Euroclone, Milan, Italy) and 10% (v/v) heat inactivated fetal bovine serum (FBS; Gibco™, Thermo Fisher Scientific, MA, USA). The MRC-5 cell line was selected for the HCoV-OC43 experiments for its high susceptibility and permissiveness to virus, as reported in literature (Min et al., 2020; Schirtzinger et al., 2022; Savoie and Lippé, 2022), and because MRC-5 cells derived from the human respiratory tract.

Human coronavirus strain OC43 (HCoV-OC43) (ATCC^®^ VR-1558) was propagated in MRC-5 cells at 34°C, using DMEM supplemented with 2% FBS (v/v) in a humidified 5% CO_2_ incubator. When full cytopathic effect developed, cultures were harvested, clarified, titrated and stocked at -70°C. Viral titration was performed by infecting MRC-5 cells with serial dilutions of viral stocks. Infected foci were detected 16 h after virus inoculum by indirect immunostaining, using a monoclonal antibody directed against the nucleoprotein of HCoV-OC43 (MAB9013, Merck KGaA, Darmstadt, Germany). Viral titers were expressed as focus forming units per mL (FFU/mL).

#### 2.3.2 Cell viability assay

Pre-seeded MRC-5 cells were treated with the drug at concentrations ranging from 1000 to 12.3 μM or equal volumes of DMSO as control, under the same experimental conditions for the antiviral assays. After incubation, the CellTiter 96 Proliferation Assay Kit (Promega, Madison, WI, USA) was used to determine the effect of lurasidone on cell viability. The effect on cell viability was expressed as a percentage, by comparing absorbances of treated cells with those of cells incubated with DMSO-treated control wells.

#### 2.3.3 Antiviral assay

The antiviral efficacy of lurasidone was determined by focus reduction assay, as described elsewhere (Milani et al., 2021). Briefly, MRC-5 sub-confluent cells in 96-well plates were infected with HCoV-OC43 at a multiplicity of infection (MOI) of 0.001 FFU/cell and concurrently treated with increasing concentrations of drug (100 to 0.12 µM). After incubation at 34°C for 16 hours, infected foci were visualized with an indirect immunocytochemistry procedure, using a primary antibody directed against the nucleoprotein of HCoV-OC43 (MAB9013, Merck KGaA, Darmstadt, Germany) or a primary antibody directed against the dsRNA (10010500, Nordic-MUbio, Susteren, Netherlands).

#### 2.3.4 Virus inactivation assay

The virucidal activity of lurasidone against HCoV-OC43 was investigated by incubating the drug at 50 µM and 10^5^ FFU of HCoV-OC43 in a 100 µL-volume for 2 h at 34°C. The virus-drug mixture was subsequently titrated, serially diluting it to the non-inhibitory concentration of lurasidone. The residual infectious viral titers were expressed as focus forming units per mL (FFU/mL).

#### 2.3.5 Time-of-addition assay

The assay evaluated the antiviral activity of lurasidone when administered before, during or after HCoV-OC43 infection. Pre-seeded cells in a 24-well plate were infected with HCoV-OC43 at MOI 0.1 and subjected to treatment with 50 µM of lurasidone for 1 hour before infection (pre-treatment), for 1 hour during virus inoculum (co-treatment) or after 1/4/18 hours from infection (post-treatment) or with an equal volume of DMSO for the untreated controls. Then, 24 hours after infection, supernatants were collected. Samples were clarified and titrated on MRC-5 cells.

#### 2.3.6 Entry assay

The assay evaluates whether the drug inhibits the entry of virus into host cells. First, a fixed inoculum of HCoV-OC43 at MOI of 0.05 was allowed to attach to prechilled confluent cells, for 20 min at 4°C. Cells were then washed with cold medium three times to remove unbound virus, treated with serial dilutions of lurasidone (from 100 to 0.4 μM), and incubated for 1 h at 34°C to allow virus entry. After incubation, outer virions were removed with a 30 second treatment of citrate buffer (pH 3.0). Then, cells were washed with warm medium three times and treated as for the antiviral assay.

#### 2.3.7 Statistical analyses

All data were analyzed with GraphPad Prism version 8.00 software. Results were expressed as mean values for three independent experiments performed in duplicate. Values of EC_50_ (half maximal effective concentration, or concentration of a compound that reduces viral infectivity by 50%), CC_50_ (half-maximal cytotoxic concentration) and standard deviation (SD) were calculated by regression analysis by fitting a variable slope-sigmoidal dose-response curve. Selectivity indexes were calculated as SI = CC_50_/EC_50_. The Student’s T-test was used to compare viral titers in virus inactivation assay. The viral titers of control and treated samples in time-of-addition assays were compared using a one-way analysis of variance (ANOVA) and a *post-hoc* Bonferroni test. Significance was set at *p* value <0.05 (*), <0.01 (**) and <0.001 (***).

### 2.4 *In vitro* PL-Pro, 3CL-Pro and RdRp inhibition assays

#### 2.4.1 Biochemical assay for SARS-CoV-2 PL-Pro measurement

The SARS-CoV-2 PL-Pro activity was measured in black 384 well plates, in 20 µl reaction buffer containing 50 mM Tris-HCl pH 7.5, 150 mM NaCl, 10% DMSO or inhibitor and 150 nM of purified protein, as described in (Paper Coluccia et al., submitted). The reaction mixture containing the enzyme was pre-incubated for 30 min with the inhibitor at 37°C. The reaction was started adding 25 µM of substrate peptide (DABCYL-FRLKGGAPIKGV-EDANS) and incubated for 10 min at RT. Products were measured with Victor Nivo (Perkin) at 320/480 (ex/em) nm. Experiments were performed in triplicate; the results report average and standard deviation of two independent replicates. Compound GRL0617 was used as positive internal control.

#### 2.4.2 Biochemical assay for SARS-CoV-2 3CL-Pro measurement

The SARS-CoV-2 nsp5 protease activity was measured in black 384 well plates (PerkinElmer), in 20 µl reaction volume containing 20 mM Tris-HCl pH 7.3, 100 mM NaCl, 1 mM EDTA, 5 mM TCEP, 0.1% BSA, 10% DMSO or inhibitor and 200 nM of purified protein (Biolatti et al., 2023). The reaction mixture containing the enzyme was pre-incubated for 30 min with the inhibitor at 37°C. The reaction was started adding 12 µM of substrate peptide (DABCYL-KTSAVLQSGFRKM-EDANS) from Bachem and incubated for 15 min at RT. Products were measured with Victor Nivo (Perkin) at 320/480 (ex/em) nm. Experiments were performed in triplicate; the results report average and standard deviation of two independent replicates. Compound GC376 was used as positive internal control.

#### 2.4.3 Biochemical assay for SARS-CoV-2 RdRp measurement

SARS-CoV-2 RdRp was expressed and purified as described (Nizi et al., 2022). The SARS-CoV-2 RdRp activity was measured in a black 96 well plate (PerkinElmer), in 25 µl reaction buffer containing 50 mM Tris HCl, 50 mM NaCl, 2.5 mM MgCl_2_, 1 mM DTT, 10% glycerol, UTP 20 µM, RNase inhibitor (20 units) pH 8.0, 0.625 µg/µl each well of PolyA and 0.03125 µg/µl well of Oligo U and 50 nM enzyme. The reaction was incubated at 37°C, in agitation at 200 rpm and a buffer PicoGreen working solution diluted in 1x Tris EDTA buffer (10 mM TrisHCl, 1 mM EDTA, pH 7.5) were added. Products were measured with Victor Nivo (Perkin) at 320/480 nm. Compound 16 was used as positive control.

### 2.5 Molecular docking

We performed an extensive docking procedure to provide an *in-silico* insight to the possible binding modes of lurasidone to the SARS-CoV-2 RBD Spike, the PL-Pro of the same virus, and the PL-Pro of HCoV-OC43. A molecular model of the Spike protein of SARS-CoV-2 has been built on the basis of PDB 6M0J (chain E); the model of the SARS-CoV-2 PL-Pro has been built on the basis of PDB 6WZU (Osipiuk et al., 2021); and a model of the HCoV-OC43 PL-Pro structure has been obtained on the basis of the sequence corresponding to positions 1,563 to 1,861 of the ORF1ab polyprotein (subsequence DKV … CLY of the reference sequence YP_009555238.1) with ColabFold (Mirdita et al., 2022), which yielded a high-confidence model (pLDDT > 0.8). The high quality of the predicted model is consistent with the availability of multiple high-homology templates, such as Nsp3 of the murine hepatitis virus (PDB: 4YPT, 64% sequence identity) and PL-Pro of the original SARS-CoV virus (PDB: 2FE8, 31% sequence identity). The lurasidone structure was obtained from PubChem (CID: 213046) and modeled with Marvin 19.4, 2019, ChemAxon (http://www.chemaxon.com) to verify its stereochemistry, add explicit hydrogens, and correct protonation for pH 7.0.

Blind docking, the process of attempting to dock without knowing the binding site, is particularly challenging and often results in false positives, especially with new targets (Corso et al., 2024). Here, we employed a procedure based on a state-of-the art diffusion generative model (Corso et al., 2022) followed by convolutional neural network-based rescoring (McNutt et al., 2021). The docking protocol consisted of a two-step process in order to increase the reliability of the results: the first step, the pose search, was carried out using DiffDock-L (online DiffDock-Web revision d134c1d), a generative model based on the diffusion modeling framework, trained to search for docked ligand poses; the second step, the docking refinement and scoring, was performed using Gnina, a convolutional neural network (CNN) trained to rescore and evaluate poses’ binding affinity. DiffDock-L outputs the 10 best-ranked poses of the ligand with the corresponding confidence score. Each of the 10 poses generated were then rescored using Gnina twice, with and without further minimization, thus providing a CNN score and affinity for the original and minimized poses. The minimization was performed starting from the DiffDock proposal, with a search space equal to its bounding box extended by 2 Å per direction.

## 3 Results

To investigate the mechanism of antiviral activity of lurasidone, ToA assays were performed on two beta-coronaviruses, SARS-CoV-2 and HCoV-OC43. ToA studies were conducted to determine the specific step(s) of the viral replication cycle affected by the drug to identify the viral target(s) involved.

### 3.1 Effect on SARS-CoV-2

To evaluate the effect of lurasidone on SARS-CoV-2, different treatment schedules were applied using a non-cytotoxic dose of the compound (Milani et al., 2021). Huh-7 cells, stably expressing the human ACE2 receptor (Huh-7-ACE2), were used in our prior study, thus chosen for experimental consistency. The cells were treated with lurasidone at a concentration of 5 µM for 1 hour, as previously determined (Milani et al., 2021). Lurasidone was then removed before SARS-CoV-2 infection (pre-treatment). Alternatively, the drug was added at the time of infection and removed when the virus inoculum was washed from the cells (co-treatment) or it was added on cells at 1, 4, 18 hours post infection (h.p.i.), in these cases the drug was added to the medium and left in the solution until the end of the experiment. The cell supernatants were harvested 24 h.p.i., and the virus titers were determined by plaque assay in Vero E6 cells.

**Figure 1:**
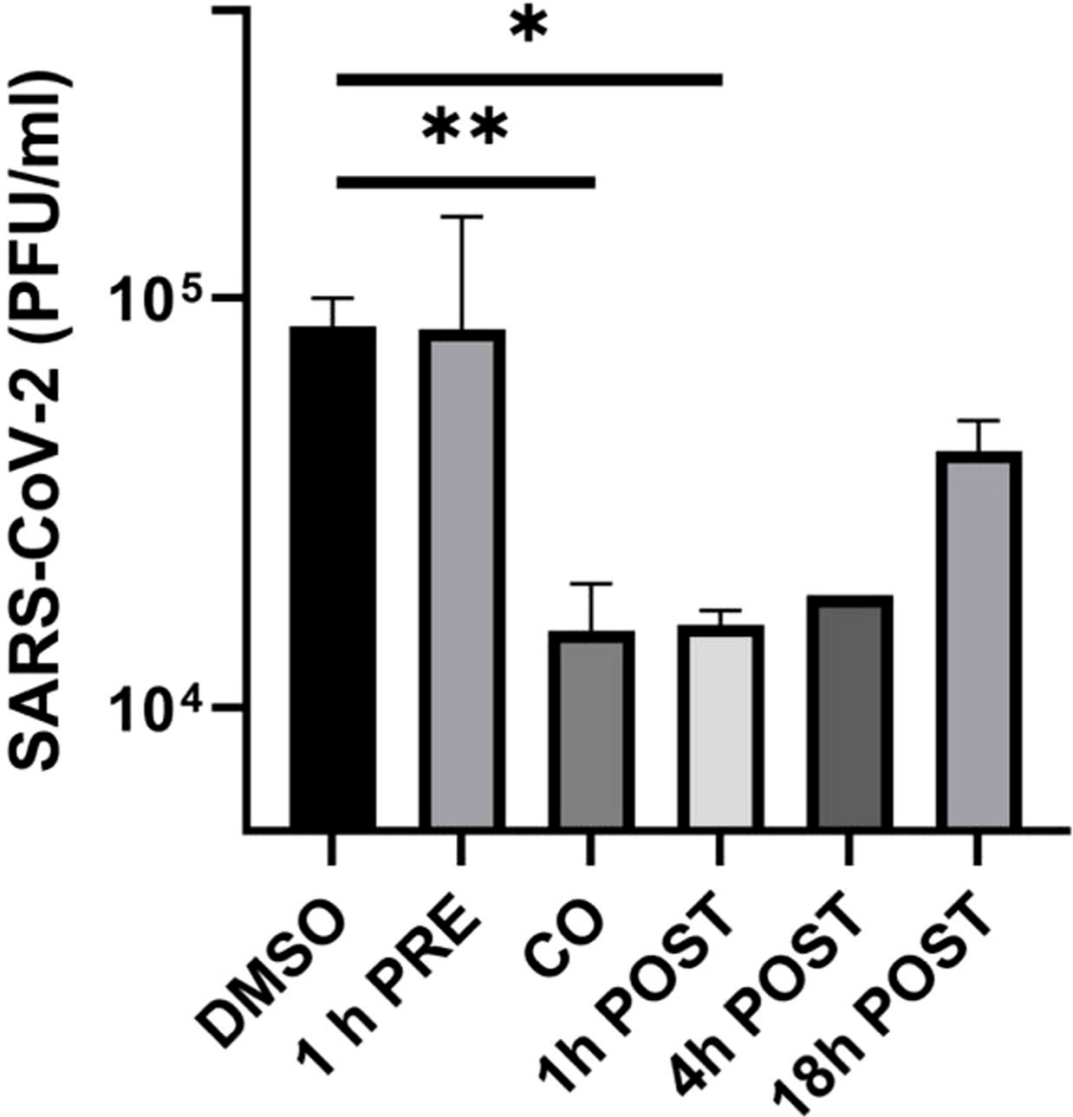
Effect of lurasidone on SARS-CoV-2 replication and inactivation. ToA experiments: 5 µM lurasidone was added to Huh-7-ACE2 cells for 1 hour before infection (1 h PRE), for 1 hour during infection (CO), or after 1, 4, 18 hours after virus inoculum (POST). Cells were treated with an equivalent volume of DMSO as a control (DMSO). Subsequently, supernatants were collected at 24 hours post-infection, and virus samples were titrated. Viral titers are expressed as mean PFU/mL and shown as average values with standard deviation and p-values, measured with a paired two-tailed t-test. Significant p-values, calculated from at least 2 independent experiments, are indicated by asterisks (**p < 0.01; *p < 0.05).

As shown in **Figure 1**, lurasidone did not affect viral replication when added to Huh-7-ACE2 cells before infection. In contrast, the inhibition of viral replication occurred when the drug was administered at the time of infection (76% inhibition compared to vehicle) or at early time of infection, showing a 78% and 79% inhibition at 1 and 4 h.p.i., respectively (**Figure 1**). A 40% inhibition was still observed when the drug was added at 18 h.p.i. These data showed that lurasidone is more active at the early steps (co-treatment and 1-4 h.p.i) of the virus replication cycle.

In addition, lurasidone (5 μM) did not show any significant virucidal activity (not shown) as demonstrated by its incubation with SARS-CoV-2 for 2 hours followed by Vero E6 cells plaque assay (see Materials and Methods).

**Figure 2:**
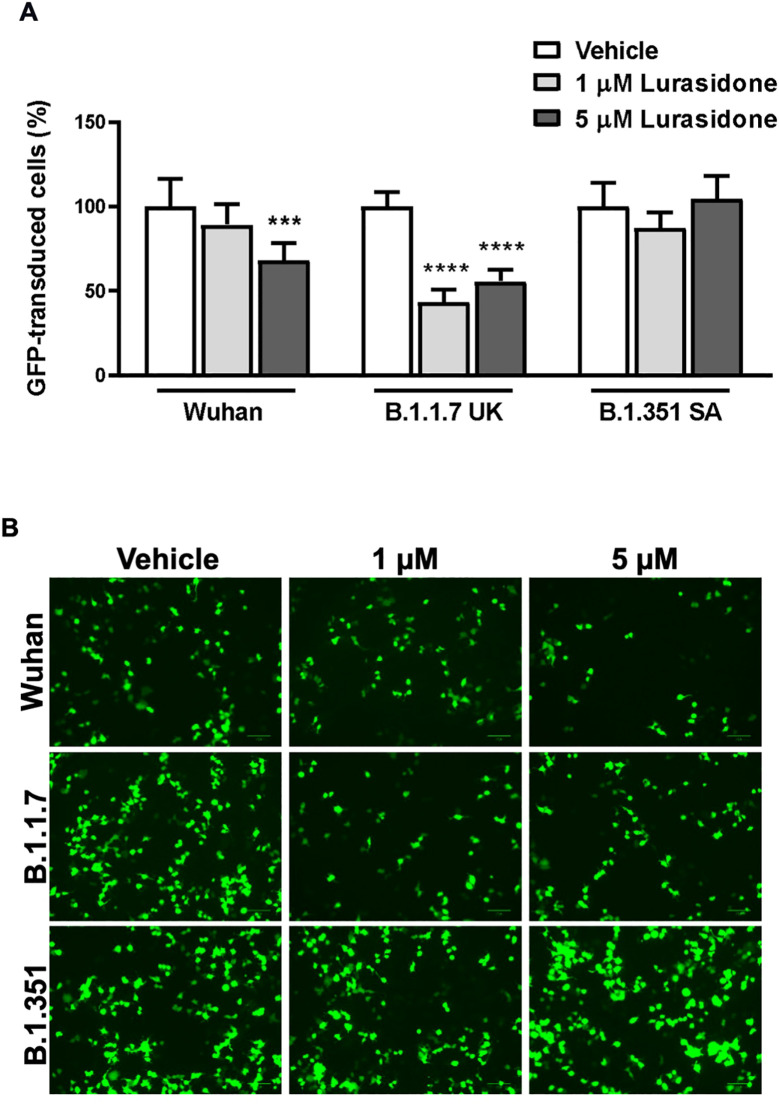
Effect of Lurasidone on pseudoviral transduction. **(A)** Percentage of GFP-positive HEK293-ACE2 cells pre-incubated for 4 h with 1 or 5 µM lurasidone and infected with pseudovirus particles exposing SARS-CoV-2 Wuhan, B.1.1.7 UK or B.1.351 South Africa (SA) Spike protein. Control cells were pre-incubated with an equivalent volume of DMSO (Vehicle). Data are the mean ± SD of GFP-positive cells compared to the control. *** p < 0.001 *vs* the corresponding vehicle according to one-way ANOVA and Bonferroni’s *post hoc* test. **(B)** Representative fluorescence microscopy images of cells treated or not with lurasidone and infected with pseudovirus displaying the different SARS-CoV-2 Spike isoforms on the envelope. Scale bar = 100 µm.

The data obtained from ToA studies suggested that lurasidone can act on early cell–virus interactions and/or on intracellular replicative cycle’s step. To investigate the ability of the drug to affect viral entry in HEK293-ACE2 cells we used pseudotyped lentiviral vectors endowed with the SARS-CoV-2 Spike glycoprotein from Wuhan, B.1.1.7 UK or B.1.351 SA (Beeg et al., 2023). Preliminary cytotoxic experiments showed that lurasidone (24 hours administration) started to reduce HEK293-ACE2 cells viability at 10 µM (~80% cell viability) and caused ~40% toxicity at the maximum tested dose of 100 µM (**Supplementary Figure S1**). Based on these results, HEK293-ACE2 cells were treated with lurasidone (1 or 5 µM) for 4 hours and then infected with the lentivirus. As shown in **Figures 2A** and **B**, 1 µM lurasidone was able to inhibit only the transduction of vectors expressing the B.1.1.7 UK Spike protein, whereas a dose of 5 µM was active also for the Wuhan isoform. On the contrary, lurasidone was not effective with the B.1.351 SA lentiviral variant at the tested concentrations, indicating dependence on the specific isoform of the SARS-CoV-2 Spike protein.

**Figure 3:**
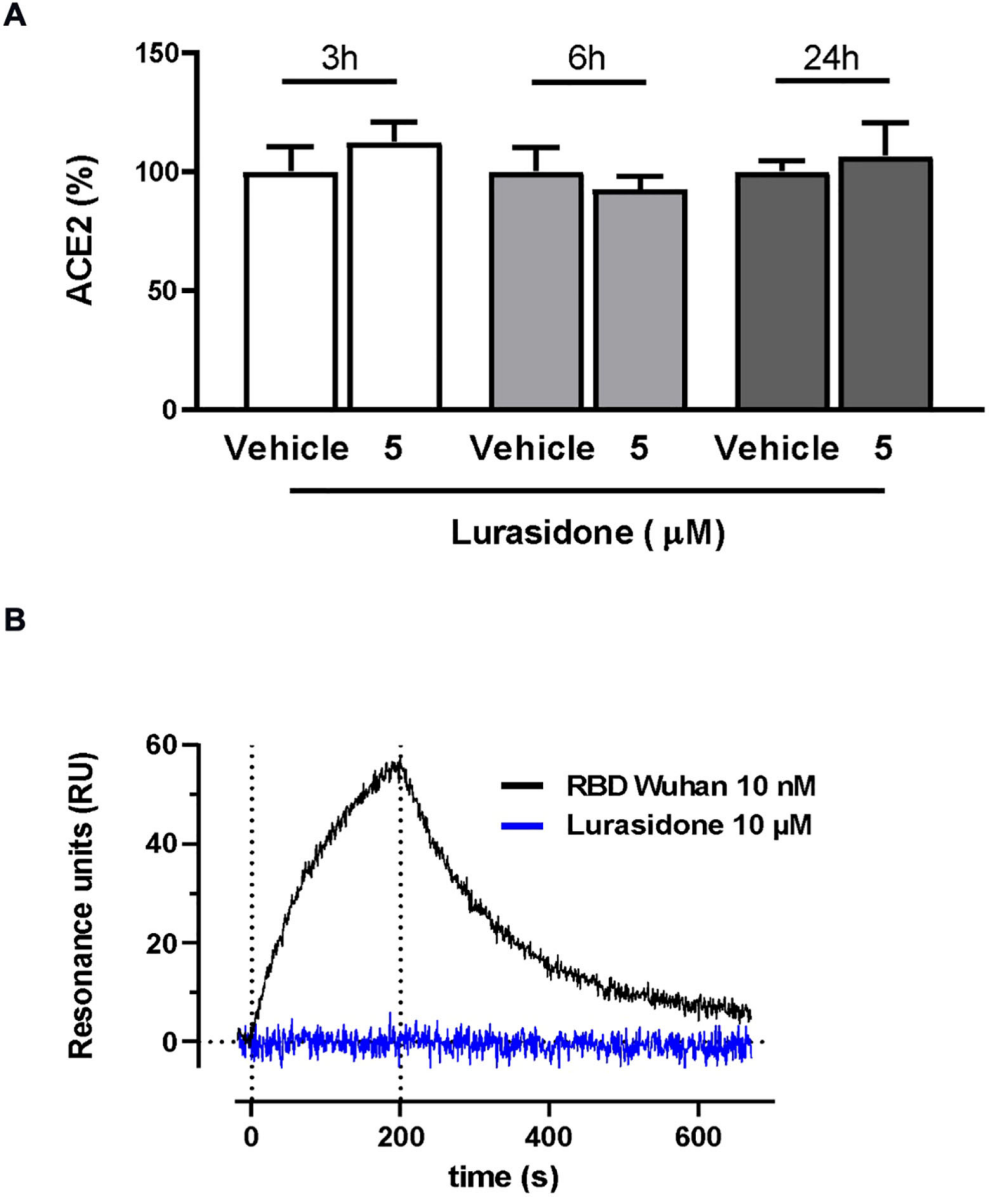
Effect of lurasidone on ACE2 expression and ACE2 binding. **(A)** Quantitative analysis of representative Western blot performed on HEK293-ACE2 cell lysates incubated for 3, 6 or 24 hours with 5 µM lurasidone. Control cells were treated with an equivalent volume of DMSO (Vehicle). ACE2 quantification is expressed as the percentage of the mean volume of the ACE2 band immunoreactivity/activity of the vehicle at the same time point. Data are the mean ± SD of three separate experiments. **(B)** Direct binding of lurasidone to rhACE2. SPR sensorgram revealed no binding signal after the injection of 10 µM lurasidone on immobilized rhACE2 (blue line) compared with the curve obtained after 10 nM SARS-CoV-2 Spike RBD Wuhan injection, used as a positive control (black line). The results shown are corrected with the data obtained on the reference channel.

The ability of lurasidone to inhibit the entry of the virus does not appear to be related to a modulation of ACE2 expression, as indicated by the different sensitivity toward different lentiviruses. In addition, Western blot analysis performed on lysates of cells treated or not for 24 h with 5 µM of the drug indicated no significant modification in the ACE2 levels (**Figure 3A**; **Supplementary Figure S2**).

The ability of lurasidone to interact directly with ACE2 was tested with surface plasmon resonance (SPR) experiments. Our results clearly indicated that the compound (10 µM) was not able to bind the recombinant human ACE2 (rhACE2) receptor immobilized on the sensor chip at difference of the Spike receptor binding domain (RBD), used as a positive control (**Figure 3B**).

### 3.2 Modeling of lurasidone binding to the SARS-CoV-2 Spike RBD

We investigated whether the different effects of lurasidone on lentivirus entry might be due to the presence of different SARS-CoV-2 Spike protein isoforms.

**Figure 4:**
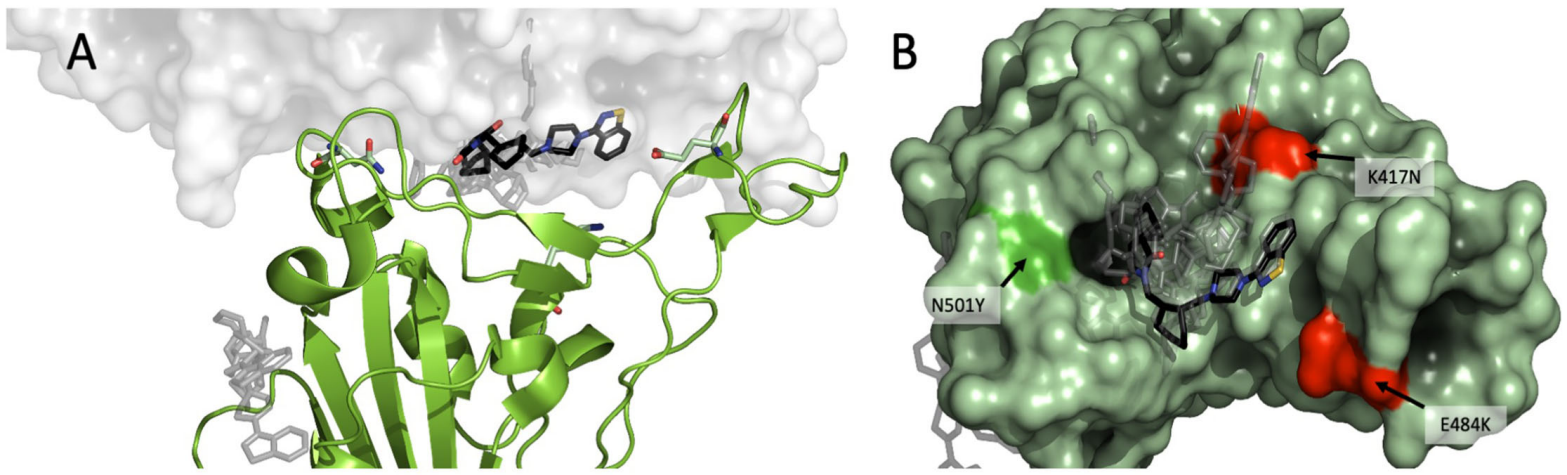
Modeling of lurasidone in the interface between Spike-ACE2. SARS-CoV-2 RBD complex (PDB 6M0J) showing **(A)** lurasidone poses docked with DiffDock-L (rank 1 in black) highlighting the putative binding site at the interface between Spike (in green) and ACE2 (in white). A secondary low-ranked site is on the side of Spike protein (gray); **(B)** overview of the putative binding site as seen from the RBD: ACE2 interface. The 10 top ranked DiffDock poses are shown, with rank 1 in black. The residues characterizing the WT, B.1.1.7 and B.1.351 SARS-CoV-2 strains are shown in red (K417 and E484) or in bright green (N501).

To analyze the possible binding site(s) of lurasidone, we built molecular models of the interface between Spike and ACE2, based on the available experimental structures. More in details, *in silico* docking was performed on the Wuhan model (PDB ID 6M0J, chain E) of the SARS-CoV-2 Spike protein first using DiffDock-L (Corso et al., 2024) then refining with Gnina (McNutt et al., 2021), as explained in the Methods section. Docked and refined poses were scored similarly, indicating a predicted affinity in the 1-10 μM range (**Supplementary Table S1**). Due to the similarity among quality scores, affinities and pose locations, for the sake of simplicity only the poses generated by DiffDock will be discussed. The results point to a binding site on the Spike RBD that is in contact with the ACE2 interface (**Figure 4A**); the site found hosts 8 out of 10 top-ranking poses, including the one with the highest confidence (two of the poses, ranked 3^rd^ and 7^th^, are found in a distal site devoid of ACE2 interaction).

A LigPlot+ diagram of the complementarity-determining interactions for the best-scoring pose is provided in **Supplementary Figure S3** (Laskowski and Swindells, 2011).

It is therefore possible to hypothesize a direct inhibition mechanism in which lurasidone binds to Spike-RBD sterically interfering with Spike-ACE2 interaction.

We compared the experimental models of Spike of Wuhan (PDB: 6M0J), B.1.1.7 (PDB: 7EKF) and B.1.351 (β) (PDB: 7V80) bound to ACE2. There are three amino-acid differences in the RBD between the three variants (**Figure 4B**), namely: B.1.1.7 differs from Wuhan for a polar-to-hydrophobic substitution N501Y (green); B.1.351 differs from B.1.1.7 for the further substitutions K417N (positive to polar) and E484K (negative to positive) (both red). Sterically, the interfaces appear to be very similar (RMSD < 1.0 Å). A remarkable feature for all the interfaces is the presence of a cavity between Spike and ACE2. Of note, the three amino acids that differentiate Wuhan RBD variant from B.1.1.7 and B.1.351 are located at about 5 Å from the docked lurasidone. Assuming that the cavity is confirmed as a binding site, it is possible to rank the amino-acid substitutions of the three variants in terms of their distance from it, namely: K417N (B.1.351) would be closest to the cavity; N501Y (B.1.1.7) would be more distant; E484K, on the contrary, would be the farthest and likely devoid of interaction. The UK ≥ Wuhan > SA susceptibility to lurasidone may be consistent with the binding pose if we assume that the K417N substitution reduces the affinity with the drug.

### 3.3 Effect on HCoV-OC43

Since HCoV-OC43 employs sialoglycan-based receptors on the cell surface, we expected a different mechanism of action of the drug independent of Spike–6ACE2 interaction.

Lurasidone antiviral efficacy against the endemic human beta-coronavirus HCoV-OC43 was already reported in our previous work (Milani et al., 2021). To further confirm the anti-HCoV-OC43 activity of lurasidone, we performed focus reduction assays by detecting two different markers of viral replication, i.e. the nucleoprotein NP and the dsRNA. Lurasidone inhibited the infection in a dose-dependent manner, with EC_50_ values around 8 µM, and CC_50_ values above 1000 µM (MTT assay) for both markers (**Table 1**), indicating that the drug affects both viral genome replication and production of viral proteins.

**Table 1:** Anti-HCoV-OC43 activity of lurasidone.

	Viral marker	EC_50_^a^ (µM) (mean ± SD ^b^)	EC_90_^c^ (μM) (mean ± SD)	CC_50_^d^ (μM)	SI^e^
Lurasidone	NP^f^	8.3 (± 2.1)	23.5 (± 4.9)	>1000	>120.5
	dsRNA^g^	8.5 (± 2.6)	11.9 (± 3.5)	>1000	>117.6

^a^Half maximal effective concentration. ^b^Standard deviation. ^c^90% effective concentration. ^d^Half maximal cytotoxic concentration. ^e^Selectivity index. ^f^Nucleoprotein. ^g^Double-strand RNA.

**Figure 5:**
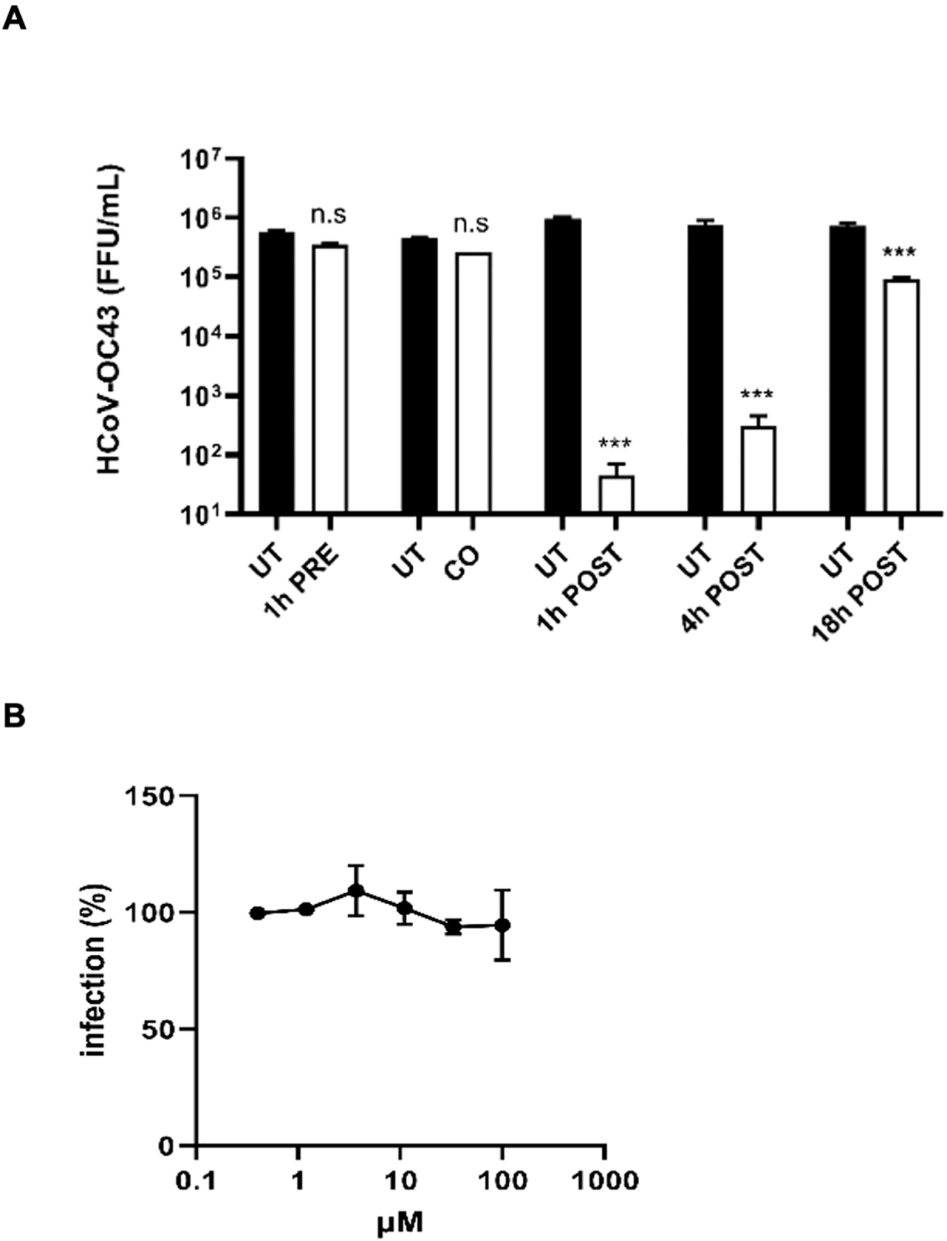
Investigation of the mechanism of action of lurasidone against HCoV-OC43. **(A)** Time-of-addition experiments. The drug at 50 µM was added to cells for 1 hours before infection (pre-treatment), for 1 hour during infection (co-treatment), or after 1-4-18 hours from virus inoculum (post-treatment); subsequently, supernatants were collected 24 hours post-infection, and virus samples were titrated. Treated and control (UT) samples were compared with one-way ANOVA. On the y-axis, viral titers are expressed as FFU/mL and shown as mean ± SEM for three independent experiments. UT, untreated. n.s., not significant. *** p < 0.001. **(B)** Entry assay. Cells were treated with serial dilutions of lurasidone (100–0.4 μM) during virus entry, and viral infectivity was assessed 16 hours post-infection by immunostaining. The percent infection (%) was calculated by comparing treated and untreated wells. Error bars represent SEMs for three independent experiments.

To investigate the mechanism of action with ToA experiments, we treated MRC-5 cells with lurasidone at the fixed dose of 50 µM (corresponding to the EC_99_ dose) before (pre-treatment), during (co-treatment), or after (1, 4, 18 hours post-treatment) infection, and we quantified the virus released in the culture supernatant 24 hours post-infection. As reported in **Figure 5A**, lurasidone was not active when added to cells before the infection (pre-treatment) or with the virus (co-treatment). On the contrary, the treatment at different times post-infection significantly inhibited virus production (post-treatment). In particular, the reduction of virus titer was most pronounced when the treatment was initiated 1 h.p.i. However, a significant reduction (p < 0.001) was still observed for the 18 hours post-infection treatment. A similar inhibitory activity was observed for the virus located intracellularly (data not shown).

As done for SARS-CoV-2, we demonstrated that lurasidone did not determine a reduction of viral titer in the treated sample as compared to the control sample, indicating that the drug was not endowed with intrinsic virucidal activity against HCoV-OC43 (not shown).

Finally, as reported in **Figure 5B**, we confirmed that lurasidone does not act during the early steps of virus infection, i.e. the entry into the host cell.

### 3.4 Effect of lurasidone on different SARS-CoV-2 proteins

To investigate whether lurasidone was targeting one of the non-structural proteins involved in the replication, we performed biochemical assays (see Materials and Methods) on purified recombinant SARS-CoV-2 RdRp, 3CL-Pro and PL-Pro proteins. As shown in **Table 2**, lurasidone inhibited PL-Pro in the submicromolar range [IC_50_ value of 0.12 µM, the protease inhibitor GLR-0617 was used as positive control (Fu et al., 2021)]. In contrast, lurasidone is inactive on both SARS-CoV-2 3CL-Pro and RdRp enzyme activities [IC_50_ > 100 µM, compounds GC376 and 16 used as positive controls (Fumagalli et al., 2023) and (Dejmek et al., 2021)] suggesting that PL-Pro represents the lurasidone Nsp target for the inhibition of SARS-CoV-2 replication.

**Table 2:** Lurasidone activity on SARS-CoV-2 PL-Pro, RdRp and 3CL-Pro enzymes.

	PL-Pro SARS-CoV-2 ^a^IC_50_ (µM)	RdRp SARS-CoV-2 ^a^IC_50_ (µM)	3CL-Pro SARS-CoV-2 ^a^IC_50_ (µM)
Lurasidone	0.12 ± 0.03	>100 (100%)^b^	>100 (87%)^b^
GRL-0617	0.30 ± 0.01	ND	ND
16	^c^ND	53 ± 2	ND
GC376	ND	ND	(1.2± 0.2)x10^-4^

a Compound concentration required to reduce enzyme activity by 50%.

b Percentage of control activity measured in the presence of indicated drug concentration.

c Not done.

### 3.5 Modeling the lurasidone binding modes to SARS-CoV-2 and OC43 PL-Pro

**Figure 6:**
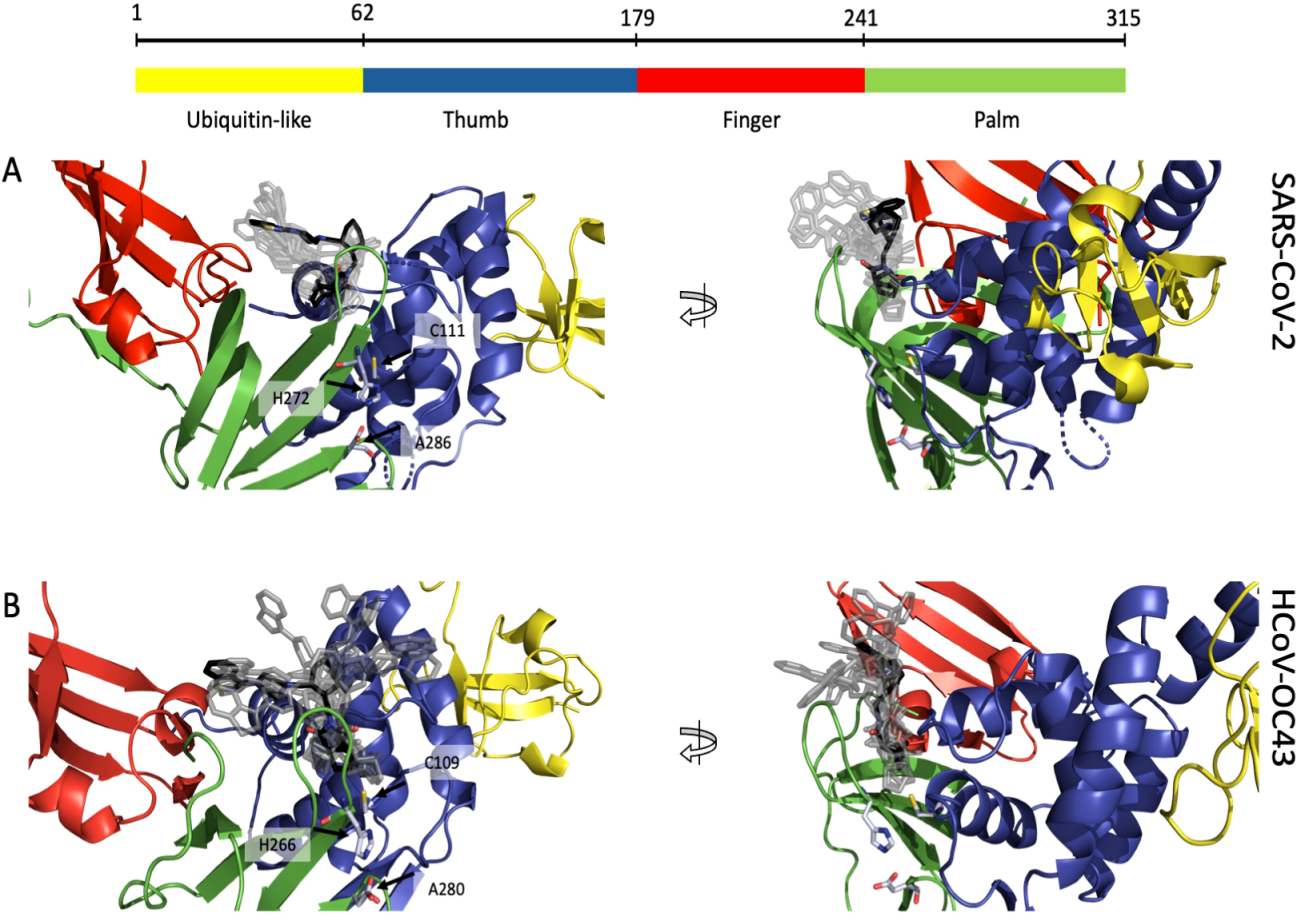
Putative binding site of lurasidone on PL-Pro proteins from SARS-CoV-2 (6WZU) and HCoV-OC43 (AF2) both colored by domain: ubiquitin-like in yellow, thumb in blue, finger in red and palm in green. **(A)** Front view (left) of SARS-CoV-2 showing lurasidone poses obtained with DiffDock (rank 1 in black) and the catalytic amino acids in light blue; side view (right) of the same structure; all the docking poses are located between the loop of the palm and the one of the thumb domains, nearby the catalytic site. **(B)** Front view (left) of HCoV-OC43 showing lurasidone poses (rank 1 in black) and the catalytic amino acids in light blue; side view (right) of the same model; again, all the docking poses are located between palm and the thumb loops, nearby the catalytic site.

We demonstrated that lurasidone has an antiviral activity against both SARS-CoV-2 and HCoV-OC43 when administered after the viral entry. Since our *in vitro* data identified the SARS-CoV-2 PL-Pro as post-entry target, due the high sequence homology of this protein among coronaviruses, we speculated that the activity against HCoV-OC43 could depend on the same enzyme. A docking procedure on PL-Pro from SARS-CoV-2 and HCoV-OC43 was performed along the same steps indicated for RBD, identifying a binding site located between the palm and the thumb domain, near the catalytic amino acids (C111 for SARS-CoV-2 and C109 for HCoV-OC43) (**Figures 6A, B**). In both proteins the ligand is in contact with a β-turn (G266-G271 in SARS-CoV-2 and I260-H266 in OC43) of the palm domain, again adjacent to the catalytic site (H272 and H266). All the 10 top-ranked poses, both original and minimized by Gnina, occupy the same pocket, and all are predicted to have an affinity in the 1-10 μM range (**Supplementary Tables S2, S3**). These results are consistent with the low μM inhibition capability found experimentally.

A comparison of the interaction fingerprints of the best-scoring pose between the two viruses is provided in **Supplementary Figure S4**.

## 4 Discussion

A dual-target antiviral drug is designed to inhibit two distinct viral mechanisms or proteins simultaneously, enhancing its effectiveness against viral infections and potentially reducing the development of drug resistance. Among them, plant-based dietary stigmastane-type saponins have shown promise as dual-target inhibitors against the SARS-CoV-2 proteases 3CL-Pro and PL-Pro (Ogunyemi et al., 2021), and Remdesivir, which is able to target viral RNA polymerase and proofreading exonuclease (Agostini et al., 2018).

Here we investigated the lurasidone’s mechanism of action identifying which replicative cycle’s step was inhibited.

On SARS-CoV-2, lurasidone is likely acting through a combination of different targets. Indeed, from ToA, the drug was already active in the co-treatment, showing that early cell–virus interactions are involved. Lurasidone does not modify the expression level of ACE2, nor does it directly interact with the host cell receptor, as confirmed with the SPR experiment. Its effect on viral entry is likely due to interference with Spike-ACE2 interaction, as demonstrated by different sensitivity of pseudotyped lentiviral vectors exposing different SARS-CoV-2 Spike isoforms, and by molecular modelling of the interface between Spike and ACE2.

Additional activity of lurasidone against SARS-CoV-2 was shown at the early times post-infection (1-4 h), when Nsps are functionally active.

An analogous strong inhibitory activity was also observed for HCoV-OC43 analyzing both the viral nucleic acid synthesis and the level of protein expression (from 1 h to 18 h post-infection). While, since HCoV-OC43 entry is independent of S-ACE2 interaction, we did not observe any antiviral activity during the viral entry. Given these results, we hypothesized a common Nsp as target in lurasidone’s action against both HCoV-OC43 and SARS-CoV-2.

From inhibition assays on different purified SARS-CoV-2 Nsps (RdRp, 3CL-Pro and PL-Pro), we demonstrated the capability of lurasidone to inhibit the activity of PL-Pro in the low µM range. Such a result was corroborated with computational studies on the interaction of the drug with both SARS-CoV-2 and HCoV-OC43 PL-Pro proteins.

Taken together, these findings underscore the versatility of lurasidone in targeting multiple stages of SARS-CoV-2 replication cycle affecting both viral entry and Nsps function. Furthermore, the capability of lurasidone to inhibit the PL-Pro, highly conserved in different coronavirus species, paves the way for further exploration of the potential therapeutic applications of this safe antipsychotic drug, which has demonstrated good tolerability in different clinical trials with minimal changes in the metabolic profile of the patients (Bawa and Scarff, 2015).

## Data Availability

The raw data supporting the conclusions of this article will be made available by the authors, without undue reservation.

## References

[B1] Agostini M. L. Andres E. L. Sims A. C. Graham R. L. Sheahan T. P. Lu X.. (2018). Coronavirus susceptibility to the antiviral remdesivir (GS-5734) is mediated by the viral polymerase and the proofreading exoribonuclease. MBio 9, 1–15. doi: 10.1128/MBIO.00221-18 PMC5844999 29511076

[B2] Bawa R. Scarff J. R. (2015). Lurasidone: A new treatment option for bipolar depression—A review. Innov. Clin. Neurosci. 12, 21. Available at: https://pmc.ncbi.nlm.nih.gov/articles/PMC4382136/. PMC4382136 25852975

[B3] Beeg M. Baroni S. Piotti A. Porta A. De Luigi A. Cagnotto A.. (2023). A comprehensive technology platform for the rapid discovery of peptide inhibitors against SARS-coV-2 pseudovirus infection. Int. J. Mol. Sci. 24, 1–12. doi: 10.3390/IJMS241512146 PMC10418426 37569522

[B4] Biolatti M. Blangetti M. Baggieri M. Marchi A. Gioacchini S. Bajetto G.. (2023). Strigolactones as Broad-Spectrum Antivirals against β-Coronaviruses through Targeting the Main Protease Mpro. ACS Infect. Dis. 9, 1310–1318. doi: 10.1021/ACSINFECDIS.3C00219/ASSET/IMAGES/LARGE/ID3C00219_0005.JPEG 37358826

[B5] Corso G. Deng A. Fry B. Polizzi N. Barzilay R. Jaakkola T. (2024). Deep confident steps to new pockets: strategies for docking generalization. Available online at: https://arxiv.org/abs/2402.18396v1. (accessed November 14, 2024).

[B6] Corso G. Stärk H. Jing B. Barzilay R. Jaakkola T. (2022). DiffDock: diffusion steps, twists, and turns for molecular docking. Available online at: https://arxiv.org/abs/2210.01776v2. (accessed November 14, 2024).

[B7] Dejmek M. Konkol’ová E. Eyer L. Straková P. Svoboda P. Šála M.. (2021). Non-nucleotide RNA-dependent RNA polymerase inhibitor that blocks SARS-CoV-2 replication. Viruses 13, 1585. doi: 10.3390/V13081585/S1 34452451 PMC8402726

[B8] Dharmarajan G. Li R. Chanda E. Dean K. R. Dirzo R. Jakobsen K. S.. (2022). The animal origin of major human infectious diseases: what can past epidemics teach us about preventing the next pandemic? Zoonoses 2, 989. doi: 10.15212/ZOONOSES-2021-0028

[B9] Diomede L. Baroni S. De Luigi A. Piotti A. Lucchetti J. Fracasso C.. (2021). Doxycycline inhibition of a pseudotyped virus transduction does not translate to inhibition of SARS-CoV-2 infectivity. Viruses 13, 1–12. doi: 10.3390/V13091745/S1 PMC8473150 34578326

[B10] Fu Z. Huang B. Tang J. Liu S. Liu M. Ye Y.. (2021). The complex structure of GRL0617 and SARS-CoV-2 PLpro reveals a hot spot for antiviral drug discovery. Nat. Commun. 12, 1–12. doi: 10.1038/s41467-020-20718-8 33473130 PMC7817691

[B11] Fumagalli V. Di Lucia P. Ravà M. Marotta D. Bono E. Grassi S.. (2023). Nirmatrelvir treatment of SARS-CoV-2-infected mice blunts antiviral adaptive immune responses. EMBO Mol. Med. 15, 1–13. doi: 10.15252/EMMM.202317580 PMC10165354 36946379

[B12] Geraghty R. J. Aliota M. T. Bonnac L. F. (2021). Broad-spectrum antiviral strategies and nucleoside analogues. Viruses 13, 667. doi: 10.3390/V13040667 33924302 PMC8069527

[B13] Kumar P. Jayan J. Kumar Sharma R. Gaidhane A. M. Syed Zahiruddin Q. Rustagi S.. (2024). The emerging challenge of FLiRT variants: KP.1.1 and KP.2 in the global pandemic landscape. QJM: Int. J. Med 117, 485–487. doi: 10.1093/QJMED/HCAE102 38867702

[B14] Laskowski R. A. Swindells M. B. (2011). LigPlot+: Multiple ligand-protein interaction diagrams for drug discovery. J. Chem. Inf. Modeling 51, 2778–2786. doi: 10.1021/CI200227U/ASSET/IMAGES/LARGE/CI-2011-00227U_0005.JPEG 21919503

[B15] Licastro D. Rajasekharan S. Dal Monego S. Segat L. D’Agaro P. Marcello A. (2020). Isolation and full-length genome characterization of SARS-coV-2 from COVID-19 cases in northern Italy. J. Virol. 94, 1–4. doi: 10.1128/JVI.00543-20 PMC7269454 32238585

[B16] McNutt A. T. Francoeur P. Aggarwal R. Masuda T. Meli R. Ragoza M.. (2021). GNINA 1.0: molecular docking with deep learning. J. Cheminformatics 13, 1–20. doi: 10.1186/S13321-021-00522-2/FIGURES/13 PMC8191141 34108002

[B17] Milani M. Donalisio M. Bonotto R. M. Schneider E. Arduino I. Boni F.. (2021). Combined in silico and *in vitro* approaches identified the antipsychotic drug lurasidone and the antiviral drug elbasvir as SARS-CoV2 and HCoV-OC43 inhibitors. Antiviral Res. 189, 1–10. doi: 10.1016/J.ANTIVIRAL.2021.105055 PMC7944860 33713730

[B18] Min J. S. Kim D. E. Jin Y. H. Kwon S. (2020). Kurarinone inhibits HCoV-OC43 infection by impairing the virus-induced autophagic flux in MRC-5 human lung cells. J. Clin. Med. 9, 2230. doi: 10.3390/JCM9072230 32674356 PMC7408680

[B19] Mirdita M. Schütze K. Moriwaki Y. Heo L. Ovchinnikov S. Steinegger M. (2022). ColabFold: making protein folding accessible to all. Nat. Methods 19, 679–682. doi: 10.1038/s41592-022-01488-1 35637307 PMC9184281

[B20] Mishchenko E. L. Ivanisenko V. A. (2022). Replication-transcription complex of coronaviruses: functions of individual viral non-structural subunits, properties and architecture of their complexes. Vavilovskii Zhurnal Genetiki i Selektsii 26, 121–127. doi: 10.18699/VJGB-22-15 35434485 PMC8983304

[B21] Niraj N. Mahajan S. Prakash A. Sarma P. Medhi B. (2022). Paxlovid: A promising drug for the challenging treatment of SARS-COV-2 in the pandemic era. Indian J. Pharmacol. 54, 452–458. doi: 10.4103/IJP.IJP_291_22 36722557 PMC10043822

[B22] Nizi M. G. Persoons L. Corona A. Felicetti T. Cernicchi G. Massari S.. (2022). Discovery of 2-Phenylquinolines with broad-spectrum anti-coronavirus activity. ACS Medicinal Chem. Lett. 13, 855–864. doi: 10.1021/ACSMEDCHEMLETT.2C00123/ASSET/IMAGES/LARGE/ML2C00123_0002.JPEG PMC9088073 35571875

[B23] Ogunyemi O. M. Gyebi G. A. Ibrahim I. M. Olaiya C. O. Ocheje J. O. Fabusiwa M. M.. (2021). Dietary stigmastane-type saponins as promising dual-target directed inhibitors of SARS-CoV-2 proteases: a structure-based screening. RSC Adv. 11, 33380–33398. doi: 10.1039/D1RA05976A 35497510 PMC9042289

[B24] Osipiuk J. Azizi S. A. Dvorkin S. Endres M. Jedrzejczak R. Jones K. A.. (2021). Structure of papain-like protease from SARS-CoV-2 and its complexes with non-covalent inhibitors. Nat. Commun. 12, 1–9. doi: 10.1038/s41467-021-21060-3 33531496 PMC7854729

[B25] Owczarek K. Szczepanski A. Milewska A. Baster Z. Rajfur Z. Sarna M.. (2018). Early events during human coronavirus OC43 entry to the cell. Sci. Rep. 8, 1–12. doi: 10.1038/s41598-018-25640-0 29740099 PMC5940804

[B26] Savoie C. Lippé R. (2022). Optimizing human coronavirus OC43 growth and titration. PeerJ 10, e13721. doi: 10.7717/PEERJ.13721/SUPP-1 35833016 PMC9272819

[B27] Schirtzinger E. E. Kim Y. Davis A. S. (2022). Improving human coronavirus OC43 (HCoV-OC43) research comparability in studies using HCoV-OC43 as a surrogate for SARS-CoV-2. J. Virological Methods 299, 1–7. doi: 10.1016/J.JVIROMET.2021.114317 PMC8500843 34634321

[B28] Teli D. Balar P. Patel K. Sharma A. Chavda V. Vora L. (2023). Molnupiravir: A versatile prodrug against SARS-coV-2 variants. Metabolites 13, 309. doi: 10.3390/METABO13020309 36837928 PMC9962121

