# Supplementary material for: The antipsychotic drug lurasidone inhibits coronaviruses by affecting multiple targets
Source: Front Cell Infect Microbiol. 2024 Nov 25;14:1487604. doi: 10.3389/fcimb.2024.1487604 (PMC11625747; doi:10.3389/fcimb.2024.1487604)
Supplement: Supplementary file 1 [file DataSheet1.pdf]

Supplementary

The antipsychotic drug lurasidone inhibits coronaviruses by affecting multiple targets

Sara Baroni[1], Tea Carletti[2], Manuela Donalisio[3], Irene Arduino[3], Irene Cazzaniga[4,5], Toni Giorgino[5], Francesca Esposito[6], Alessia Porta[1], Luisa Diomede[1], Ada De Luigi[1], Marco Gobbi[1], David Lembo[3], Alessandro Marcello[2], Enzo Tramontano[6], Mario Milani[5], Eloise Mastrangelo[4,5,*]

[1]*Department of Molecular Biochemistry and Pharmacology, Istituto di Ricerche Farmacologiche Mario Negri IRCCS, Via Mario Negri 2, 20156 Milano, Italy*

[2]*Laboratory of Molecular Virology, International Centre for Genetic Engineering and Biotechnology, Padriciano 99 I-34149 Trieste, Italy*

[3]*Dipartimento di Scienze Cliniche e Biologiche, Università di Torino, Regione Gonzole, 10 I-10043 Orbassano (Turin), Italy*

[4]*Dipartimento di Bioscienze, Università degli Studi di Milano, via Celoria 26, 20133 Milano, Italy*

[5]*Consiglio Nazionale delle Ricerche, Istituto di Biofisica, Via Celoria 26, 20133 Milano, Italy*

[6]*Department of Life and Environmental Sciences, University of Cagliari, S.P. 8 Monserrato, Sestu Km 0.700 – I-09042 Monserrato, Italy*

*Address correspondence to:

Dr. Eloise Mastrangelo,

CNR, Istituto di Biofisica,

Via Celoria 26, I-20133, Milano, Italy.

e-mail: eloise.mastrangelo@cnr.it

The supplementary materials concern:

Figure 1S. Dose-response effect of Lurasidone on cell viability.

Figure 2S. Effect of lurasidone on ACE2 expression and ACE2 binding.

Figure 3S. Interaction diagram between lurasidone and WT SARS-CoV-2 RBD.

Figure 4S. Interaction diagrams between lurasidone and PLPro of SARS-CoV-2 (black) and HCoV-OC43 (grey).

Table 1S. Detailed metrics for the binding of lurasidone with WT spike protein.

Table 2S. Detailed metrics for the binding of lurasidone with SARS-CoV-2 PL-Pro.

Table 3S. Detailed metrics for the binding of lurasidone with OC43 PL-Pro.

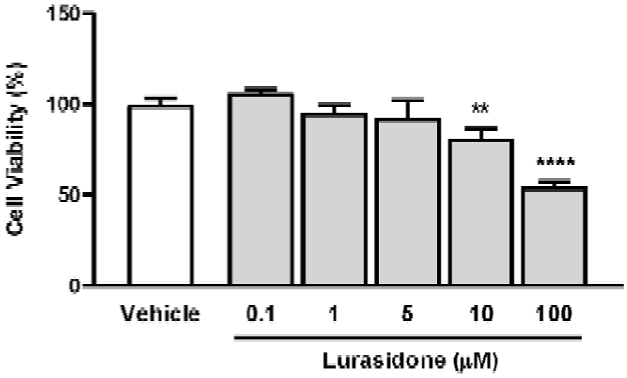

Figure 1S. Dose-response effect of Lurasidone on cell viability. HEK293-ACE2 treated with increasing concentrations of Lurasidone, in the MTT assay. Data are reported as the mean ± SD percentage of viable cells compared to control cells (treated with vehicle only). ** $p \leq 0.001$ and **** $p < 0.0001$ vs. vehicle according to one-way ANOVA and Bonferroni's post hoc test.

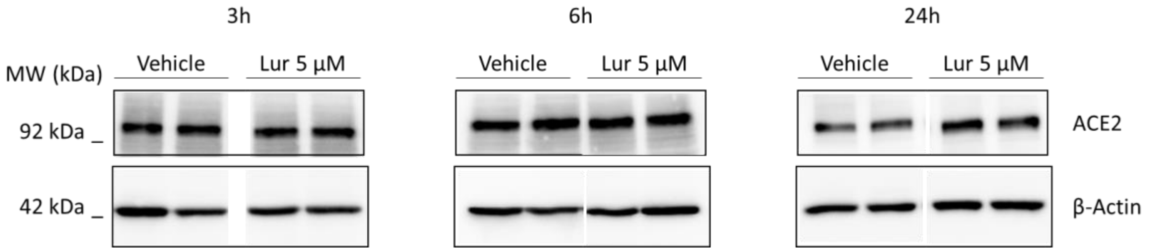

Figure 2S. Effect of lurasidone on ACE2 expression. ACE2 expression in lysates of HEK293-ACE2 cells incubated for 3, 6 or 24 hours with 5 μM lurasidone. Control cells were treated with an equivalent volume of DMSO (Vehicle). Equal amount of proteins was loaded in each gel lane (10 μg) and immunoblotted with anti-ACE2 or anti-β-actin primary antibody. Signals were detected using HRP-coupled secondary antibodies and revealed with a ChemiDoc Touch Imaging System. Western blot images are representative examples of three separate experiments.

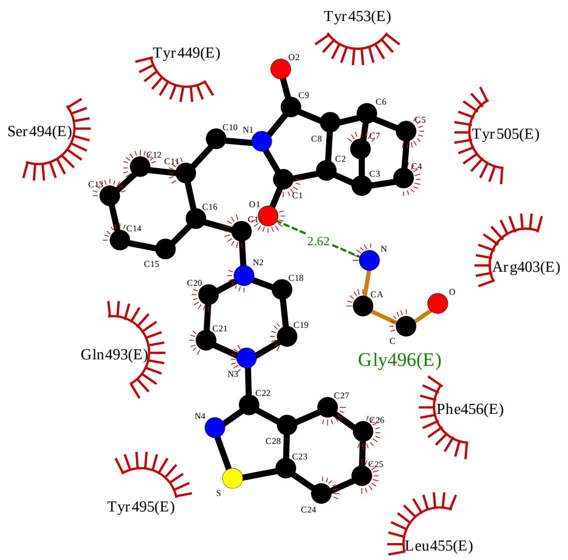

Figure 3S. **Interaction diagram between lurasidone and WT SARS-CoV-2 RBD** (PDB:6M0J). Red: hydrophobic contacts; green: hydrogen bonds. Diagram produced with LigPlot+.

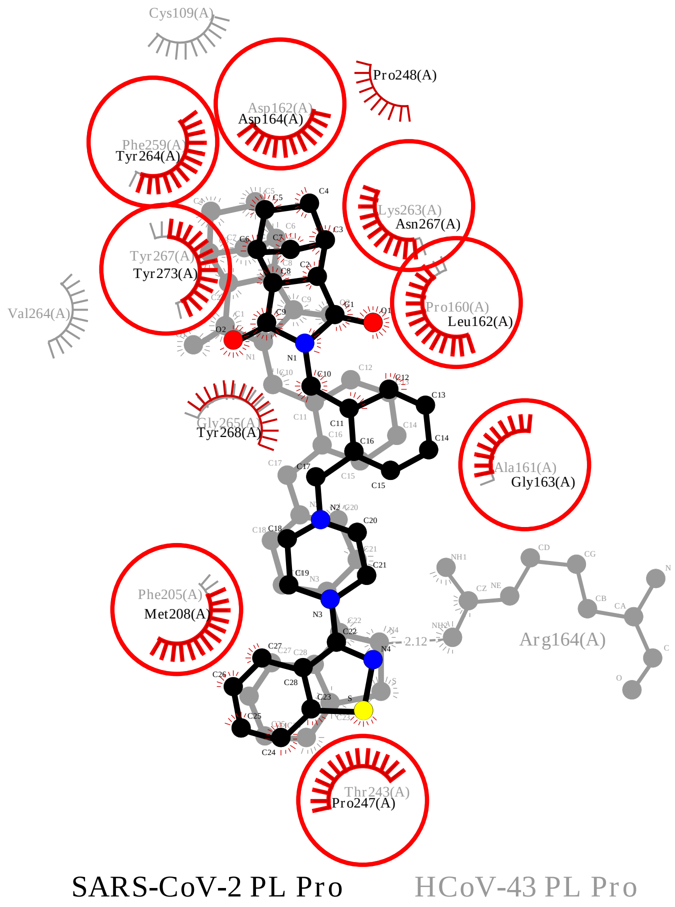

Figure 4S. **Interaction diagrams between lurasidone and PL-Pro of SARS-CoV-2 (black) and HCoV-OC43 (grey).** Red: hydrophobic contacts; green: hydrogen bonds. Conserved interactions are circled. Diagram produced with LigPlot+.

Table 1S. Detailed metrics for the binding of lurasidone with WT spike protein. Score metrics obtained from a two-step docking with DiffDock-L and Gnina. Legend – Diffdock confidence: measure of the quality of the Diffdock prediction (c > 0 good, -1.5 < c < 0 moderate, c < -1.5 bad); CNN score: value from 0 to 1 (1 being best) determining the quality of the pose; CNN VS: is the product of CNN affinity and CNN score; CNN affinity: quality of the binding, measured as pK values; CNN affinity var: measure of the uncertainty of the predicted CNN affinity; Affinity: quality of the binding, measured as kcal/mol; RMSD: measure of the displacement between the two methods. Minimized: same as before but for the minimized poses.

Diffdock rank	Diffdock confidence	CNN score	CNN VS	CNN affinity	CNN affinity var	Minimized CNN score	Minimized CNN VS	Minimized CNN affinity	Minimized CNN affinity var	Minimized Affinity	Minimized RMSD
1	-1.72	0.34	1.86	5.53	1.11	0.41	2.24	5.50	0.77	0.42	3.32
2	-1.86	0.04	0.21	4.99	0.70	0.29	1.47	5.16	0.34	2.33	3.41
3	-2.32	0.39	2.01	5.22	0.63	0.36	1.88	5.27	0.12	-4.30	1.60
4	-2.8	0.31	1.80	5.71	1.03	0.34	1.96	5.77	0.47	-4.52	2.75
5	-3.11	0.30	1.64	5.54	1.02	0.20	1.11	5.50	0.17	-4.89	3.58
6	-4.2	0.27	1.34	4.98	0.81	0.57	3.29	5.81	1.50	-2.57	4.27
7	-4.21	0.41	2.15	5.27	0.79	0.37	1.93	5.17	0.24	-4.50	2.76
8	-4.27	0.13	0.55	4.21	1.43	0.38	2.22	5.86	1.51	-3.86	3.35
9	-4.57	0.11	0.49	4.56	0.50	0.50	2.88	5.80	0.64	-4.09	3.76
10	-4.99	0.41	2.13	5.14	0.40	0.16	0.85	5.36	0.03	-5.31	4.05

Table 2S. Detailed metrics for the binding of lurasidone with SARS-CoV-2 PL-Pro. See the legend of Table 1S for column interpretation.

Diffdock rank	Diffdock confidence	CNN score	CNN VS	CNN affinity	CNN affinity var	Minimized CNN score	Minimized CNN VS	Minimized CNN affinity	Minimized CNN affinity var	Minimized Affinity	Minimized RMSD
1	-1.27	0.03	0.18	5.17	0.42	0.51	3.00	5.92	0.77	-3.68	6.72
2	-1.51	0.04	0.18	4.92	0.43	0.56	3.17	5.67	0.64	-0.18	7.65
3	-2.24	0.03	0.14	4.93	0.18	0.51	2.84	5.60	1.02	-1.63	6.09
4	-2.36	0.02	0.09	5.33	0.82	0.30	1.53	5.15	0.41	-2.21	5.90
5	-2.4	0.01	0.07	5.29	0.71	0.25	1.40	5.53	0.52	-4.22	5.60
6	-2.41	0.03	0.18	5.28	0.40	0.09	0.49	5.54	0.40	-5.21	5.33

7	-2.42	0.05	0.27	5.06	0.23	0.50	2.91	5.84	0.83	-2.59	5.71
8	-2.65	0.03	0.15	4.93	0.28	0.46	2.60	5.64	0.68	-3.17	4.22
9	-3.02	0.04	0.17	4.65	0.29	0.07	0.40	5.44	0.31	-3.74	7.08
10	-3.57	0.06	0.30	4.80	0.29	0.13	0.75	5.69	1.07	-4.48	5.96

Table 3S. Detailed metrics for the binding of lurasidone with OC43 PL-Pro. See the legend of Table S1 for column interpretation.

Diffdock rank	Diffdock confidence	CNN score	CNN VS	CNN affinity	CNN affinity var	Minimized CNN score	Minimized CNN VS	Minimized CNN affinity	Minimized CNN affinity var	Minimized Affinity	Minimized RMSD
1	-1.34	0.22	1.24	5.56	0.61	0.48	2.65	5.56	0.51	-4.85	3.42
2	-1.56	0.35	1.96	5.60	0.54	0.46	2.71	5.85	1.01	1.21	2.34
3	-1.75	0.05	0.28	5.30	0.30	0.36	1.94	5.43	0.32	-3.76	2.73
4	-2.05	0.21	1.20	5.60	0.61	0.23	1.26	5.41	0.46	-5.21	2.55
5	-2.13	0.38	2.10	5.58	0.33	0.54	3.29	6.14	0.27	-1.61	2.86
6	-2.34	0.11	0.60	5.34	0.22	0.14	0.78	5.64	0.90	-6.93	3.85
7	-2.63	0.33	1.76	5.36	0.26	0.37	2.20	5.95	2.34	-3.43	7.19
8	-2.84	0.27	1.39	5.20	0.27	0.31	1.70	5.50	0.16	-3.89	2.98
9	-3.39	0.13	0.65	5.07	0.14	0.45	2.61	5.84	0.86	-3.95	2.98
10	-3.67	0.15	0.76	5.04	0.37	0.18	0.95	5.32	0.05	-5.64	3.61
